# Health technology assessment of biosimilars worldwide: a scoping review

**DOI:** 10.1186/s12961-020-00611-y

**Published:** 2020-08-26

**Authors:** Bruna de Oliveira Ascef, Ana Carolina de Freitas Lopes, Patrícia Coelho de Soárez

**Affiliations:** Preventive Medicine Department, Faculty of Medicine, The University of São Paulo, Av. Dr. Arnaldo, 455, sala 2228, São Paulo, SP CEP: 01246-903 Brazil

**Keywords:** Biosimilar pharmaceuticals, Technology assessment, Biomedical, Health policy, Evidence-based decisions

## Abstract

**Background:**

Health technology assessment (HTA) should provide an assessment of a technology’s effects on health and of the related social, economic, organisational and ethical issues. HTA reports on biosimilars can specifically assess their immunogenicity, their extrapolation to one or more conditions, and the risks of interchangeability and substitution. We aimed to complete a scoping review within the context of HTA organisations to synthesise HTA reports on biosimilars and to map the extension, scope and methodological practices.

**Main body:**

A scoping review methodology was applied. The sources for biosimilars HTA reports were database searches and grey literature from HTA organisation websites up to June 2019. HTA reports of biosimilars were classified as full HTA, mini-HTA or rapid reviews. Data were extracted and recorded on a calibrated predefined data form. We identified 70 HTA reports of biosimilars of 16 biologic products (65.71% in 2015–2018) produced by 13 HTA organisations from 10 countries; 2 full HTAs, 4 mini-HTAs and 64 rapid reviews met the inclusion criteria. Almost all the rapid reviews gave no information regarding any evidence synthesis method and approximately half of the rapid reviews did not appraise the risk of bias of primary studies or the overall quality of evidence. All full-HTAs and mini-HTAs addressed organisational, ethical, social and legal considerations, while these factors were assessed in less than half of the rapid reviews. The immunogenicity and extrapolation of one or more conditions were often considered. The majority of full-HTAs and mini-HTAs contained an assessment of switching and a discussion of an educational approach about biosimilars. No HTA report rejected the adoption/reimbursement of the biosimilar assessed.

**Conclusion:**

HTA of biosimilars are emerging in the context of HTA organisations and those that exist often duplicate reports of the same biosimilar. Most HTA reports of biosimilars do not conduct a systematic literature review or consider economic issues. No report has rejected the adoption/reimbursement of biosimilars. There is a need to standardise the minimum criteria for the development of HTA on biosimilars to ensure a better understanding and better decision-making.

## Background

A biosimilar is a biological drug highly similar to and with no clinically meaningful differences from an existing approved reference biologic [1]. Biosimilars are approved through stringent and clearly defined regulatory processes after having undergone rigorous analytical, immunogenic, non-clinical and clinical comparative evaluations [2, 3]. The biosimilar approval pathways are similar in most countries when their goal is to prove biosimilarity [4, 5]. However, different countries tend to have different regulations to decide substitution and interchangeability [1, 4–8].

Due to their structural complexity and production in living systems under strictly controlled conditions, biologicals are expensive to develop and manufacture [9]. Consequently, treatment costs are usually high, with a huge impact on health systems budgets [10]; it is estimated that biologics account for over a quarter of spending on pharmaceuticals [11]. Health systems should benefit from biosimilars as they lead to price competition that improves patient access to safe and effective biological medicines and help health systems with restricted budgets [12]. Despite this, the use of biosimilars, the cost-savings achieved and their market share vary considerably between biosimilar drugs, therapeutic areas, countries and even within the same country [6, 12, 13]. Those differences can be linked to an overall lack of biosimilar familiarity worldwide accompanied by concerns about biosimilars efficacy, safety, immunogenicity, extrapolation, switching and interchangeability [8, 14–16].

The vital contribution of Health Technology Assessment (HTA) for informed decision-making and the creation of equitable, high-quality and sustainable healthcare systems is well recognised and consolidated in scientific and technological practice [17]; however, there is still no generally accepted position on its utilisation in relation to biosimilars [18]. In fact, the evidence needed to obtain marketing authorisation from a registration authority does not always correspond to the data requirements for an HTA [9, 18]. HTAs provide a systematic evaluation of a technology’s potential direct and indirect effects on health in addition to an assessment of its social and ethical implications and the organisational requirements for its application and are aimed mainly at informing decision-making regarding health technologies [19, 20]. With regards to biosimilars, HTA reports face a number of additional challenges such as assessment of the drug in relation to immunogenicity, interchangeability, and substitution, consideration of the extrapolation for one or more conditions, and the inclusion of discussion of an educational approach designed to inform the public about biosimilars [9, 18].

A variety of institutes, units and organisations of HTA in universities, hospitals or in governmental and non-governmental bodies conduct HTA worldwide. They differ substantially in many dimensions, including how they understand and use HTA, their regulatory power and their independence. However, they have a common objective, that is, to produce HTAs to inform a variety of health decision-making processes or to assess the added value of health technologies and develop a recommendation to inform decision-making at the local or national level, pricing and/or reimbursement decision-making, re-assessment of practices, or to inform clinicians, providers, and patients about the proper use of healthcare interventions [21–24].

As there is no information regarding studies that systematically reviewed how HTA organisations are producing HTA reports of biosimilars worldwide, our main objective was therefore to complete a scoping review within the context of HTA organisations to synthesise HTA reports that assessed biosimilars. Specifically, we aimed to map (1) the frequency and type of HTA reports on biosimilars (full-HTA, mini-HTA or rapid review) and when they were produced; (2) which countries and organisations produced HTA reports of biosimilars; (3) the main biosimilars analysed; (4) the key methodological characteristics found by the type of HTA; and (5) the key methodological characteristics that are specific to the assessment of biosimilars.

## Methodology

### Study design

We used a scoping review methodology to map HTA reports discussing biosimilars and produced by HTA organisations. This scoping review was developed using the methodological framework proposed by Arksey and O’Malley [25] and refinements by the Joanna Briggs Institute [26].

### Protocol

The Preferred Reporting Items for Systematic Reviews and Meta-analysis Extension for Scoping Reviews (PRISMA-ScR) [27] was used to develop our protocol (Additional file 1).

### Inclusion and exclusion criteria

For the systematisation of the evidence, a structured question was developed in the population, concept, and context framework.

#### Population

Any type of population with no restrictions regarding age, gender, health condition and any other key demographic features.

#### Concept

A biosimilar or similar biotherapeutic product is defined by WHO as a biotherapeutic product that is similar in terms of quality, safety and efficacy to an already licensed reference biotherapeutic product [2]. It will only be considered biosimilar if the product has undergone rigorous regulation and a comparability exercise, i.e. head-to-head comparison of a biotherapeutic product with a licensed originator product to establish similarity in quality, safety and efficacy [2]. Only biosimilar drugs analysed as an intervention were considered eligible.

#### Context

HTA agencies or organisations has been created mainly due to the need to have an instance with the specific purpose of informing policy-makers about the development, dissemination and use of health technologies [28]. This scoping review considers HTA reports of biosimilars in the context of HTA organisations or agencies or other bodies worldwide. We considered HTA reports identified from the websites of HTA organisations listed as members on the following leading global HTA networks: (1) the International Network of Agencies for Health Technology Assessment (INAHTA); (2) Health Technology Assessment International; (3) the European Network for Health Technology Assessment; and (4) *Rede de Avaliação de Tecnologia em Saúde das Américas* or those identified on the specific databases.

We only considered HTA reports of biosimilars produced by HTA organisations and classified as full HTA, mini-HTA or rapid review by the criteria of the classification system devised by Merlin et al. to be eligible for the review [29]. Reports classified as ‘others’ were excluded.

### Study selection for HTA

The identification of HTA reports was divided into two main sources, namely (1) a comprehensive search of specific databases related to the topic and (2) a manual search for HTA reports on the main websites of HTA organisations (grey literature). No restrictions regarding language or publication year were made.

#### Comprehensive database search for reports related to the topic

Two review authors (BOA and ACFL) independently identified studies through systematic searches in specific electronic databases related to the HTA, namely the Center for Review and Dissemination Database, including the HTA database and the NHS Economic Evaluation Database. To build the search strategy, we used MeSH (‘Biosimilar Pharmaceuticals’[Mesh]) and text words (biosimilar or follow on biologics). No restrictions on the publication year were made. The search was performed up to May 2019. We updated the search on 03 June 2019 and incorporated the reports found into the review.

Two authors independently evaluated the titles and abstracts identified through the search strategy. Studies that did not meet the inclusion criteria defined previously were excluded. Any disagreement in the selection of the studies was settled by consensus. The full-length articles were downloaded and the same two authors read the full-text articles. In cases of disagreements, a third reviewer (PCS) was consulted.

#### Manual search for HTA reports in the main websites of HTA organisations or agencies (grey literature)

The manual search of these sources was developed in four phases, as follows: (1) two reviewers (BOA and ACFL) manually searched the publicly accessible member lists of the main HTA network websites (INAHTA, Health Technology Assessment International, the European Network for Health Technology Assessment and *Rede de Avaliação de Tecnologia em Saúde das Américas*) to identify the websites of the HTA organisations worldwide. (2) Between 6 and 10 August 2018, on each webpage of an HTA organisation identified, we searched for HTA reports on biosimilars. We updated this search on 03 June 2019 and all new reports found were incorporated. No restrictions on the publication year of HTA reports on biosimilars were made. Two reviewers (BOA and ACFL) searched all the webpages of HTA organisations for any document with the word ‘biosimilar’. This search was made in according to the language of origin of the HTA organisation/agency. Any document containing the term biosimilar was considered for the identification of the studies. All full-text documents were downloaded and saved with an ID, agency name or organisation, language, and URL of the website. The total number of documents found on each website was recorded. (3) Two reviewers (BOA and ACFL) screened all the documents found on each website for eligibility. Studies that did not meet the inclusion criteria defined previously were excluded. The reasons for excluding them were recorded. Any disagreements were resolved through a consensus of the two reviewers. (4) One reviewer (BOA) read the full texts again. If the report was eligible, the reviewer classified all reports according to the criteria described by Merlin et al. [29]. A second reviewer (ACFL) rescreened and reclassified a random sample of 10% of the excluded articles. Any disagreements were resolved through a consensus of the two reviewers. In the case of frequent and/or substantial disagreements, a verification process for any excluded articles was planned. As there were no disagreements, the verification process was not employed. In the case of uncertainty regarding inclusion/exclusion and the HTA classification criteria, a third reviewer was involved (PCS). The main reason for excluding any texts was recorded.

### Defining types of HTA reports and charting data

The types of HTA were divided into four categories according to the criteria of the classification system devised by Merlin et al. [29], namely full HTA, mini-HTA, rapid review and ‘other’. A report was classified as a full HTA if it met at least seven out of the eight criteria, whereas a mini-HTA report had to meet at least criteria 1, 2, 4, 6 and 7 and rapid reviews had to meet at least criteria 1 and 2. Any reports that did not fit into any of the three preceding categories were classified as ‘other’ and were considered ineligible for this scoping review (Additional file 2).

One team member (BOA) independently extracted and recorded data on a predefined data form validated by the other members (BOA and ACFL) (Table 1).

**Table 1:** Scoping review charting form

Charting dimensions	Aspects
General information	
General information about HTA organisations and their HTA report	Name and abbreviation of HTA organisation Country of origin of HTA organisation Year of publication Type of biosimilars (substance active, brand name, product name) Biologic reference (brand name) Identification of anatomical and therapeutic group of biosimilars ATC Code (therapeutic drug groups) and ATC^a^ 1st level - anatomical main group)
Methodological characteristics of biosimilar HTA report	
Criterium 1	Described the characteristics and current use of the technology (Yes/No) *1. Yes, very detailed (detailed description, including drug use (indications, pharmaceutical form, and dosage), what is biosimilar and their originator), regulatory status and other information* *2. Yes, but not detailed (brief description, no information provided about biosimilar or drug use, or another relevant biosimilars’ information)* *3. Not described*
Criterium 2	Evaluated safety and effectiveness issues (Yes/No) *1. Assessment of efficacy/effectiveness and safety* *2. Assessment of efficacy/effectiveness only* *3. Assessment of safety only* *4. Assessment of efficacy/effectiveness and safety but evidence was not found* *5. Neither safety nor effectiveness are evaluated*
Criterium 3	Did they conduct an economic analysis? *1. Yes, a cost-minimisation analysis* *2. Yes, a cost-utility analysis* *3. Yes, a cost-effectiveness analysis* *4. Yes, a cost-benefit analysis* *5. Not clear* *6. No, they did not*
Criterium 4	Provided information on costs/financial impact *1. They provided information on costs and a BIA* *2. They provided information on costs, but they did not provide a BIA* *3. No information provided on costs or BIA*
Criterium 5	Discussed organisational considerations *1. Yes* *2. No*
Criterium 6	Conducted a comprehensive systematic literature review or a systematic review of high-level evidence *1. Yes, a systematic review of high-level evidence was conducted* *2. Yes, a comprehensive systematic literature review was conducted* *3. No, there is no evidence that a systematic review was conducted*
Criterium 7	Critically appraiseed the quality of the evidence base *1. Yes, adequately (they assessed the risk of bias of primary studies and/or the overall quality of evidence with appropriate tools/approach)* *2. Yes, partially (they critically appraised the risk of bias of primary studies and/or the overall quality of evidence, but they did not report the use of any tools/approach)* *3. Neither appraised the risk of bias of primary studies or the overall quality of evidence* *4. No (evidence not found)*
Criterium 8	Addressed ethical, social and legal considerations *1. Yes* *2. No*
Particularities of HTA reports of biosimilars	
Item 1	Immunogenicity was considered *1. Yes* *2. No*
Item 2	Risk of switching or interchangeability was considered? *1. Yes* *2. No*
Item 3	Extrapolation was considered? *1. Yes* *2. No*
Item 4	Do they mention any educational approach about biosimilars to patients, clinicians or pharmacists? *1. Yes* *2. No*
Item 5	Statement in favour or against adoption or reimbursement of biosimilar or no statement *1. Statement in favour of adoption/reimbursement of biosimilar* *2. Statement against adoption/reimbursement of biosimilar* *3. No statement in favour or against biosimilars*

*ATC* Anatomical, Therapeutic and Chemical, *BIA* budget impact analyses, *HTA* health technology assessment

^a^Each biosimilar was coded according to the ATC Classification from WHO, 2020 [30]

## Results

### Selection of sources of evidence

In total, we searched 136 websites of HTA organisations. Of these, 50 HTA organisation websites had at least one document discussing biosimilar content. In the screening phase, 258 documents were excluded and the reasons for exclusion were (1) documents/studies that mentioned but did not perform an assessment of biosimilars (*n* = 74); (2) documents that were informative and educational about biosimilars (*n* = 54); (3) documents related to the regulatory status of biosimilars (*n* = 45); (4) duplicates (*n* = 26); (5) narrative reviews/overviews/editorials of biosimilars (*n* = 25); (6) documents on the use of a specific biosimilar (indications, use, forms, adverse effects) (*n* = 9); (7) consensus or society positions about biosimilars (*n* = 7); (8) a list of biosimilars available in the health system (*n* = 6); (9) guidelines on how to assess biosimilars (*n* = 5); (10) standard forms to submit for the assessment of biosimilars (*n* = 4); and (11) primary studies of biosimilars (*n* = 3).

**Fig. 1:**
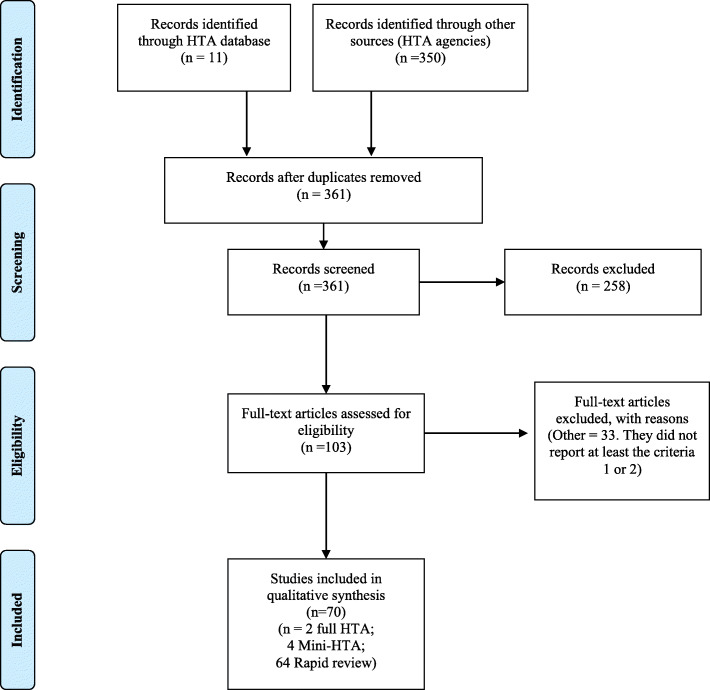
Flow diagram for HTA of biosimilars. *HTA* health technology assessment

In the eligibility phase, 103 documents were considered as potential HTA reports of biosimilars and were classified by the criteria of Merlin et al. [29]. We identified 70 HTA reports on biosimilars and 33 documents classified as ‘others’ were excluded. Of the 70 HTA reports on biosimilars included, 2 (2.86%) were classified as full HTA and 4 (5.71%) as mini-HTA, whereas the majority were classified as rapid reviews (91.43%) (Fig. 1).

### General information about HTA reports on biosimilars

Regarding the HTA organisations identified to produce HTA reports of biosimilars, we found that 13 HTA organisations from 10 countries produced the reports (Table 2). *Haute Autorité de Santé*, a French HTA organisation, produced half of the rapid reviews, whereas HTA organisations in Canada, the United Kingdom and Italy were responsible for producing around 41% of the rapid reviews. Moreover, all the mini-HTAs were from Canada, mostly from the Canadian Agency for Drugs and Technologies in Health. With regards to the full HTAs, one was from the National Institute for Health and Care Excellence (NICE) in the United Kingdom and the other from the *Institut national d’excellence en santé et en services* in Canada.

**Table 2:** HTA reports of biosimilars by organisations, countries of origin and type of reports

HTA organisation	Country of HTA organisation	Full-HTA (*n* = 2)	Mini-HTA (*n* = 4)	Rapid reviews (*n* = 64)
HAS	France	0	0	31
CADTH	Canada	0	3	9
INESSS	Canada	1	1	7
NIHR HC	United Kingdom	0	0	3
NICE	United Kingdom	1	0	1
RER	Italy	0	0	4
Veneto CRUF	Italy	0	0	1
AQuaAS	Spain	0	0	2
TLV	Sweden	0	0	1
AHTAPol	Poland	0	0	1
ACE	Singapore	0	0	2
ICER	United States	0	0	1
IECS	Argentine	0	0	1

*ACE* Agency for Care Effectiveness; *AHTAPol* Agencja Oceny Technologii Medycznych; *AQuaAS* Agència de Qualitat i Avaluació Sanitàries de Catalunya; *CADTH* Canadian Agency for Drugs and Technologies in Health; *HAS* Haute Autorité de Santé; *HTA* health technology assessment; *ICER* Institute for Clinical and Economic Review; *IECS* Instituto de Efectividad Clinica Sanitaria; *INESSS* Institut national d’excellence en santé et en services; *NICE* National Institute for Health and Care Excellence; *NIHR HC* The National Institute for Health Research Horizon Scanning Centre; *RER* Regione Emilia-Romagna; *TLV* Dental and Pharmaceutical Benefits Agency; *Veneto CRUF* Regione del Veneto, Coordinamento Regionale Unico sul Farmaco

**Fig. 2:**
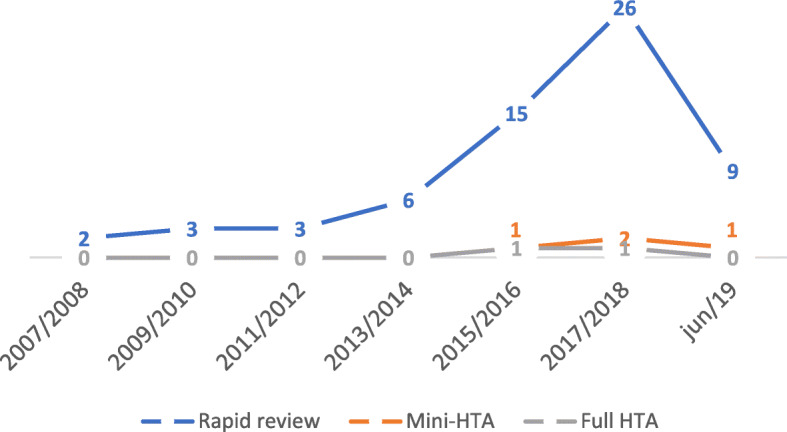
Production of HTA reports of biosimilars from 2007 to June 2019. *HTA* health technology assessment

Figure 2 illustrates the number of HTA reports of biosimilars (rapid review, mini-HTA and full HTA) that were produced over 12 years and 5 months. HTA report production followed a fairly similar pattern over the first 8 years evaluated, with only a small amount of rapid reviews produced. Most of the reports (46 of 70 reports or 65.71%) were produced between 2015 and 2018.

Table 3 shows the type and frequency of biosimilars assessed in the HTA reports by ATC (Anatomical, Therapeutic and Chemical) classification first level (anatomical main group). In total, we identified biosimilars of 16 active substances from 4 main anatomical groups. Biosimilars that are antineoplastic and immunomodulating agents were the majority (72.85%). In this group, tumour necrosis factor-alpha (TNF-α) inhibitors (biosimilars of etanercept, infliximab and adalimumab) were the most frequent type evaluated (35.71%). Infliximab was the most frequently assessed biosimilar, accounting for 17 (24.29%) of the HTA reports, followed by biosimilars of pegfilgrastim (10%) and of insulin glargine and trastuzumab (both 8.57%) (Table 3). We identified seven different biosimilar brand names for five filgrastim biosimilars and five different biosimilar brand names for three infliximab biosimilars (Table 3). The most frequent biosimilar assessed in the HTA reports according to the brand name was Inflectra® (*n* = 11; 15.72%), data not shown.

**Table 3:** HTA reports by ATC 1st level, anatomical main group, brand and product name of biosimilar

ATC Code	ATC – active substance (Chemical subgroup)	Number of HTA reports (%)	Biosimilar - brand name [product name]
A (Alimentary tract and metabolism) 6 (8.57%)			
A10AE04	Insulin glargine (Insulins and analogues for injection, long-acting)	6 (8.57%	Abasaglar [LY2963016]; Basaglar [LY2963016]
B (Blood and blood-forming organs) 10 (12.85%)			
B01AB05	Enoxaparin (Heparin group)	4 (5.71%)	Abenox Enoxaparin Becat [Crusia-AFT]; Crusia [Crusia-AFT]; Inhixa
B03XA01	Erythropoietin (Other antianaemic preparations)	5 (7.14%)	Retacrit [SB309]; Binocrit [X575]; Abseamed [X575];
G (Genito-urinary system and sex hormones) 2 (2.86%)			
G03GA05	Follitropin alfa (Gonadotropins)	2 (2.86%)	Ovaleap [XM17]; Bemfola [AFOLIA-150];
H (Systemic hormonal preparations^a^) 3 (4.29%)			
H01AC01	Somatropin (Somatropin and somatropin agonists)	2 (2.86%)	Omnitrope
H05AA02	Teriparatide (Parathyroid hormones and analogues)	1 (1.43%)	Movymia [RGB-10]
L (Antineoplastic and immunomodulating agents) 50 (71.42%)			
L01XC02	Rituximab (Monoclonal antibodies)	4 (5.71%)	Rixathon [GP2013]; Truxima [CT-P10]; Novex
L01XC03	Trastuzumab (Monoclonal antibodies)	6 (8.57%)	Ogivri [Myl 1401O]; Trazimera [PF-05280014]; Ontruzant [SB3]; Kanjinti [ABP 980]; Herzuma [CT-P6]
L01XC07	Bevacizumab (Monoclonal antibodies)	2 (2.86%)	Mvasi [ABP 215]
L03AA13	Pegfilgrastim (Colony stimulating factors)	7 (10.00%)	Lapelga Fulphila [MYL-1401H]; Pelmeg [B121019]; Ziextenzo [LAEP2006]; Pelgraz [MYL-1401H];
L03AA02	Filgrastim (Colony stimulating factors)	5 (7.14%)	Biograstim [G-CSF] Ratiopharm [G-CSF]; Ratiograstim [XM02]; Tevagrastim [XM02]; Zarzio [EP2006]; Nivestim [Filgrastim-aaf]; Grastofil [Apo-filgrastim]
L03AX13	Glatiramer acetate (Other immunostimulants)	1 (1.43%)	Glatect
L04AB01	Etanercept (TNF-α inhibitors)	4 (5.71%)	Benepali [SB4]; Brenzys [SB4]; Erelzi [GP2015]
L04AB02	Infliximab (TNF-α inhibitors)	17 (24.29%)	Remsima [CT-P13]; Inflectra [CT-P13]; Renflexis [SB2]; Flixabi [SB2]; Zessly [PF-06438179/GP1111]
L04AB04	Adalimumab (TNF-α inhibitors)	4 (5.71%)	Hulio [FKB327]; Hyrimoz [GP2017]; Amgevita [ABP 501]; Imraldi [SB5]

*HTA* health technology assessment; *TNF-α* tumour necrosis factor-alpha

^a^Systemic hormonal preparations, excluding sex hormones and insulins

### Methodological characteristics of biosimilar HTA reports

Table 4 shows the results of the charting of the HTA reports on biosimilars. The two full HTAs met all eight criteria, including a discussion of ethical, social and legal considerations, which is optional. These reports also included a discussion of organisational considerations. Both full HTAs provided information on costs, but only one provided a budget impact analysis (BIA). They also critically appraised the risk of bias of primary studies and/or the overall quality of evidence, but they did not report the use of any methodological tools to evaluate this.

**Table 4:** Results of biosimilar HTA report charting

HTA classification criteria	Full HTA (*n* = 2)	Mini-HTA (*n* = 4)	Rapid review (*n* = 64)
Criterium 1 – Description of the characteristics and current use of the technology			
1. Yes, very detailed	0 (0.0%)	3 (75.0%)	31 (48.4%)
2. Yes, but not detailed	2 (100.0%)	1 (25.0%)	33 (51.6%)
Criterium 2 – Evaluation of safety and effectiveness issues			
1. Assessment of efficacy/effectiveness and safety	2 (100.0%)	4 (100.0%)	53 (82.8%)
3. Assessment of safety only	0 (0.0%)	0 (0.0%)	4 (6.2%)
4. Assessment of efficacy/effectiveness and safety but evidence was not found	0 (00.0%)	0 (00.0%)	7 (11.0%)
Criterium 3 – Did they conduct an economic analysis?			
1. Yes, a cost-minimisation analysis	2 (100.0%)	0 (0.0%)	5 (7.8%)
6. No, they did not	0 (0.0%)	4 (100.0%)	59 (92.2%)
Criterium 4 – Provision of information on costs/financial impact			
1. They provided information on costs and a BIA	1 (50.0%)	3 (75.0%)	24 (37.5%)
2. They provided information on costs but they did not provide a BIA	1 (50.0%)	1 (25.0%)	6 (9.4%)
3. Neither they provided information on costs nor a BIA	0 (0.0%)	0 (0.0%)	34 (53.1%)
Criterium 5 – Discussion of organisational considerations			
1. Yes	2 (100.0%)	3 (75.0%)	18 (28.1%)
2. No	0 (0.0%)	1 (25.0%)	46 (71.9%)
Criterium 6 – Conducted a comprehensive systematic literature review or a systematic review of high-level evidence			
1. Yes, a systematic review of high-level evidence was conducted	1 (50.0%)	0 (0.0%)	0 (00.0%)
2. Yes, a comprehensive systematic literature review was conducted	1 (50.0%)	4 (100.0%)	2 (3.1%)
3. No, there is no information regarding any method of the evidence synthesis	0 (0.0%)	0 (0.0%)	62 (96.9%)
Criterium 7 – Critically appraised the quality of the evidence base			
1. Yes, adequately	0 (0.0%)	3 (75.0%)	0 (0.0%)
2. Yes, partially	2 (100.0%)	1 (25.0%)	27 (42.2%)
3. Neither appraised the risk of bias of primary studies or the overall quality of evidence	0 (0.0%)	0 (0.0%)	30 (46.9%)
4. Not applicable (evidence not found)	0 (0.0%)	0 (0.0%)	7 (10.9%)
Criterium 8 – Addressed ethical, social and legal considerations			
1. Yes	2 (100.0%)	4 (100.0%)	25 (39.1%)
2. No	0 (0.0%)	0 (0.0%)	39 (60.9%)

*BIA* budget impact analysis; *HTA* health technology assessment

The four mini-HTAs conducted a comprehensive systematic literature review and three assessed the risk of bias of primary studies and/or the overall quality of evidence with an appropriate methodological approach. Furthermore, three of the mini-HTAs provided information on costs and a BIA and discussed organisational considerations. All four mini-HTAs addressed ethical, social and legal considerations (Table 4).

The majority of the rapid reviews (96.9%) did not conduct a systematic review of the clinical evidence and approximately 47% did not appraise the risk of bias of primary studies or the overall quality of evidence. Five (7.8%) rapid reviews included a cost-minimisation analysis and 24 (37.5%) provided information on costs and a BIA. However, these reports did not include criteria 6 and 7 in their reports and were not classified as full or mini-HTAs. Less than 40% of rapid reviews addressed organisational, ethical, social and legal considerations (Table 4).

### Particularities of HTA reports of biosimilars

Concerning the particularities of HTA reports of biosimilars, the assessment of immunogenicity was provided by 1 full HTA, 4 mini-HTAs, and 39 of the 64 (60.93%) rapid reviews (Table 5).

**Table 5:** Key methodological characteristics of HTAs of biosimilars

HTA reports	Immunogenicity was considered	Risk of switching/interchangeability was considered	Extrapolation of one or more condition was considered	Educational approach about biosimilars was considered	Statement in favour, against or not declared about the adoption/reimbursement of biosimilar		
					Statement in favour	Statement against	Not declared
Full HTA (*n* = 2)	1	1	1	1	2	0	0
Mini-HTA (*n* = 4)	4	4	4	4	4	0	0
Rapid review (*n* = 64)	39	13	42	14	51	0	13

*HTA* health technology assessment

All mini-HTAs and one full HTA addressed the risk of switching or interchangeability, extrapolation of one or more conditions, and provided an educational approach about biosimilars to patients, clinicians or pharmacists. Only 13 of 64 (20.31%) rapid reviews considered the risk of switching or interchangeability and 14 of 64 (21.87%) provided an educational approach about biosimilars to patients, clinicians or pharmacists. Approximately 66% of the rapid reviews considered the extrapolations of biosimilars to more than one condition (Table 5).

No report rejected the adoption or reimbursement of the biosimilar assessed. All the mini-HTAs and full HTAs made a statement in favour of the adoption or reimbursement of the biosimilar assessed. Around 20% of the rapid reviews did not provide a final statement about the adoption/reimbursement of the biosimilars (Table 5).

## Discussion

The scoping review identified 70 HTA reports for biosimilars of 16 biologic products (65.71% in 2015–2018) produced by 13 HTA organisations from 10 countries. Rapid reviews were the main type of biosimilar HTA report found. Full HTAs and mini-HTAs were rare. The majority of rapid reviews did not give any information regarding any evidence synthesis method and approximately half of the rapid reviews did not appraise the risk of bias of primary studies or the overall quality of evidence. All full-HTAs and mini-HTAs addressed organisational, ethical, social and legal considerations but these criteria were assessed in less than half of the rapid reviews. Immunogenicity was often assessed in the HTA reports for biosimilars. Additionally, extrapolation of one or more conditions was often considered. On the other hand, the assessment of switching or interchangeability and an educational approach about biosimilars were present mostly in the mini-HTAs and full HTAs. No HTA reports for biosimilars rejected the adoption/reimbursement of the biosimilar assessed.

An increasing number of assessments of biosimilars was found in line with the increasing number of biosimilars reaching the market. Allocati et al. [12] reported that, up to July 2019, the European Medicines Agency had approved 55 biosimilars of 16 biologic products, mostly between 2017 and 2019. This agrees with the results of this scoping review, which found that most of the HTA reports were produced between 2017 and 2018. A rich pipeline with over 240 biosimilars in development will mean that launches will be coming with increasing frequency [11]. However, it is not possible to know if the HTA reports of biosimilars will follow this pattern.

Few countries are producing HTA reports for biosimilars. A French HTA agency produced half of the rapid reviews and all the mini-HTAs were produced by Canadian organisations. Only one report was from Latin America. Although patients in emerging markets, such as Latin America, stand to gain the greatest increase in access as a result of biosimilar competition, they typically have relatively low access to biologic medicines compared to developed markets and they put significant effort into encouraging the use of copy-biologics, which have not gone through a biosimilar pathway with strict regulatory scrutiny such as the biosimilar guidelines for the European Medicines Agency, the United States Food and Drug Administration or WHO [11].

The majority of biosimilars assessed were antineoplastic and immunomodulating agents such as TNF-α inhibitors (biosimilars of etanercept, infliximab and adalimumab), followed by insulins. These drugs represent the three largest biologic therapy areas, worth $110 billion, over half of all biologic global revenue [11]. They account for 9 of the top 10 biologics in the global market [11]. Health economic evaluations and budget impact studies of biosimilars of these agents can lead to significant cost savings and these studies have shown that biosimilars of TNF-α inhibitors are cost-effective in the perspectives of the health systems that assessed them [31–37].

As in this scoping review, Allocati et al. [12], in their study about marketing authorisation of biosimilars in Europe, found that, in many cases, several biosimilars of the same biologics are licensed by the same or different marketing authority under different commercial names, even when the active molecule (and the relevant pivotal trial) is the same. Allocati et al. could not find a plausible scientific or regulatory reason for this and suggested that it may be due to different local legal requirements across countries in Europe or for commercial reasons; however, this situation can cause confusion between HTA agencies and a duplication of work.

Our findings indicate that there are only a limited number of robust and complete HTA reports for biosimilars, mainly due to the absence of a systematic literature review, failures in the appraisal of the quality of the evidence, and a lack of consideration of economic issues such as budget impact and cost-effectiveness. A summary of the top 10 challenges to produce an HTA identified by INAHTA members pointed out that the increasing demand for HTAs was often accompanied by a request for greater speed, leading to an increased demand for rapid HTA [17]. Additionally, INAHTA members considered that the use of an evidence grading system when formulating recommendations was a challenge [17]. This is reflected in the findings of this scoping review, since the majority of reports did not adequately appraise the quality of evidence with an appropriate approach or tools [38] such as the GRADE system for assessment of overall quality of evidence or tools to assess the methodological quality of primary studies such as the Assessing the Methodological Quality of Systematic Review (AMSTAR) and the Cochrane Risk of Bias tools. Although health economic evaluations and BIA are widely recognised and available for use in studies, they were not common practice in the HTA reports of biosimilars found in this scoping review [38, 39].

Organisational, social and ethical issues, such as organisational drug access, pharmacovigilance, patient support programmes, ethical considerations of equity, and expert and public consultations, were often addressed on full HTAs and mini-HTAs, while, on rapid reviews, these issues were not often assessed. The literature shows that some of the assessments considered as context-dependent aspects (i.e. organisational, social, cultural, political, legal and ethical factors that reflect the linkage between HTA and health policy practice) are still not widely assessed on HTA [20, 39–41]. It is important to stress that, if HTAs do not include consideration of these context-dependent issues, only clinical evidence will remain as the main source of information for decision-makers and HTAs will fail to establish a robust bridge over this particular knowledge–action gap [20]. In other words, HTA reports of biosimilars may fail to be implemented and used by decision-makers since they would not consider the context in which it would be implemented and used in healthcare practice [42]. For instance, if a hypothetical biosimilar HTA report informed on the efficacy and safety of the treatment but did not inform on whether there were any consultations to health systems stakeholders about imposing the automatic switching of biologics to biosimilars, how would the doctors assess the trustworthiness of prescribing biosimilars? How would patients know and trust the biosimilars that they are being prescribed? This could lead to a low rate of acceptance of biosimilars. However, public consultation and educational programmes about switching, for example, should be considered in HTA reports.

A rapid review of the literature shows that one of the main barriers to access to biosimilars comes from health professionals who are not convinced by expert opinion in regard to treatment interchangeability and substitution, and the perception that there is a lack of data in relation to efficacy and safety [43]. In addition, as another review pointed out, the idea that a biosimilar’s approval can be based on extrapolation from other indications has not been fully accepted and has led to a reluctance to use biosimilars [4]. Even though extrapolation was often considered in HTA reports on biosimilars, only one-quarter of the reports addressed the risks of substitution and considered educating clinicians, patients and pharmacists about biosimilars.

This scoping review included 70 HTA reports for biosimilars (2 full HTAs, 4 mini-HTAs and 64 rapid reviews) mostly produced between 2015 and 2018, showing that that 13 HTA organisations from 10 countries have produced HTA reports of biosimilars for 16 active substances. Biosimilars that are antineoplastic and immunomodulating agents were the majority (72.85%), including TNF-α inhibitors. The majority of rapid reviews did not give any information regarding any evidence synthesis methods and approximately half of the rapid reviews did not appraise the risk of bias of primary studies or the overall quality of evidence. All full-HTAs and mini-HTAs addressed organisational, ethical, social and legal considerations but these criteria were assessed in less than half of the rapid reviews. Immunogenicity assessment and extrapolation of one or more conditions were often considered in all reports. The assessment of switching or interchangeability and an educational approach about biosimilars were present mostly in the mini-HTAs and full HTAs. No report has rejected the adoption of the biosimilar assessed.

Furthermore, our findings pointed out that there is a duplication of HTA reports of the same biosimilar, suggesting that there is a lack of collaboration between HTA organisations [44] and there is a clear need to standardise the minimum criteria of a biosimilar HTA report to allow health systems to be able to make more informed decisions about adopting biosimilars.

With the findings of this scoping review, our intention is to stimulate a critical reflection and to lead policy-makers, decision-makers and stakeholders to improve their way of producing and using HTA reports of biosimilars for their health systems. This study should be useful for HTA organisations to adopt or adapt principles of rigorous, comprehensive and transparent HTAs as well as to motivate HTA organisations to adopt a common knowledge strategy for evidence-informed decision-making to avoid duplicity. Furthermore, patients, practitioners in health and decision-makers should be aware of the information that is being given in those HTA reports of biosimilars that can sometimes partly respond to a question to make an evidence-based decision. Thus, they should use HTA reports of biosimilars to enhance the trust in biosimilars; however, this requires a thorough understanding of HTA features and potential [45].

The main limitation of this scoping review was the source of evidence, which relied mostly on the grey literature. An additional limitation was having a single member of the team in charge of the recording and classification of the HTAs although a second member checked a random sample. Furthermore, inaccuracy may have been introduced when data were extracted from HTA reports not available in English or not in a language spoken by those who extracted the data. In these cases, it was necessary to rely on information from translation tools.

## Conclusion

Few HTA organisations are producing HTAs for biosimilars and there is duplicated work. Although most reports have supported the adoption of biosimilars, these statements have often been based on reports lacking a systematic literature review and not considering economic issues, using tools such as health economic evaluations and BIA. It is vital to develop standardised minimum methodological criteria for HTAs for biosimilars that will support a better understanding of the public regarding the information provided by an HTA report on biosimilars as well as for better decision-making in health systems when deciding to adopt or reimburse biosimilars.

## Supplementary information

**Additional file 1.** Preferred Reporting Items for Systematic reviews and Meta-Analyses extension for Scoping Reviews (PRISMA-ScR) Checklist [27].

**Additional file 2. **Classification of HTA reports according to the criteria of Merlin et al. [29]. *HTA* Health Technology Assessment. * A synthesis that collates all empirical evidence fitting pre-specified eligibility criteria to answer a specific research question. Systematic reviews are conducted according to a pre-specified protocol. The methods used are selected to minimise bias, thus providing more reliable findings from which conclusions can be drawn and decisions made.

## Abbreviations

ATC: Anatomical, Therapeutic and Chemical
BIA: Budget impact analysis
HTA: Health technology assessment
INAHTA: International Network of Agencies for Health Technology Assessment
NICE: National Institute for Health and Care Excellence
TNF-α: Tumour necrosis factor-alpha
WHO: World Health Organization
