# Supplementary material for: Health technology assessment of biosimilars worldwide: a scoping review
Source: Health Res Policy Syst. 2020 Aug 26;18:95. doi: 10.1186/s12961-020-00611-y (PMC7448328; doi:10.1186/s12961-020-00611-y)
Supplement: Supplementary file 2 — Additional file 2. Classification of HTA reports according to the criteria of Merlin et al. [29]. HTA Health Technology Assessment. * A synthesis that collates all empirical evidence fitting pre-specified eligibility criteria to answer a specific research question. Systematic reviews are conducted according to a pre-specified protocol. The methods used are selected to minimise bias, thus providing more reliable findings from which conclusions can be drawn and decisions made. [file 12961_2020_611_MOESM2_ESM.docx]

| **Criteria** | **Description** | **Full HTA** | **Mini-HTA** | **Rapid Review** | **Other** |
| --- | --- | --- | --- | --- | --- |
| **1** | Describe the characteristics and current use of the technology | Always | Always | Always | Always |
| **2** | Evaluate safety and effectiveness issues | Always | Always | Always | Optional |
| **3** | Determine the cost-effectiveness of the technology through economic modeling (when it is appropriate) | Always | Not  performed | Not  Performed | Not  performed |
| **4** | Provide information on costs/financial impact | Always | Always | Optional | Optional |
| **5** | Discuss organizational considerations | Always | Optional | Optional | Optional |
| **6** | Conduct a comprehensive systematic literature review* or a systematic review of high-level evidence | Always | Always | Optional | Optional |
| **7** | Critically appraise the quality of the evidence base | Always | Always | Optional | Optional |
| **8** | Address ethical, social and legal considerations | Optional | Optional | Optional | Optional |
